# BioModels—15 years of sharing computational models in life science

**DOI:** 10.1093/nar/gkz1055

**Published:** 2019-11-08

**Authors:** Rahuman S Malik-Sheriff, Mihai Glont, Tung V N Nguyen, Krishna Tiwari, Matthew G Roberts, Ashley Xavier, Manh T Vu, Jinghao Men, Matthieu Maire, Sarubini Kananathan, Emma L Fairbanks, Johannes P Meyer, Chinmay Arankalle, Thawfeek M Varusai, Vincent Knight-Schrijver, Lu Li, Corina Dueñas-Roca, Gaurhari Dass, Sarah M Keating, Young M Park, Nicola Buso, Nicolas Rodriguez, Michael Hucka, Henning Hermjakob

**Affiliations:** 1 European Molecular Biology Laboratory, European Bioinformatics Institute (EMBL-EBI), Wellcome Trust Genome Campus, Hinxton, Cambridge CB10 1SD, UK; 2 Babraham Institute, Babraham Research Campus, Cambridge CB22 3AT, UK; 3 California Institute of Technology, Pasadena, 91125, CA, USA; 4 State Key Laboratory of Proteomics, Beijing Proteome Research Center, Beijing Institute of Lifeomics, National Center for Protein Sciences (The PHOENIX Center, Beijing), Beijing 102206, China

## Abstract

Computational modelling has become increasingly common in life science research. To provide a platform to support universal sharing, easy accessibility and model reproducibility, BioModels (https://www.ebi.ac.uk/biomodels/), a repository for mathematical models, was established in 2005. The current BioModels platform allows submission of models encoded in diverse modelling formats, including SBML, CellML, PharmML, COMBINE archive, MATLAB, Mathematica, R, Python or C++. The models submitted to BioModels are curated to verify the computational representation of the biological process and the reproducibility of the simulation results in the reference publication. The curation also involves encoding models in standard formats and annotation with controlled vocabularies following MIRIAM (minimal information required in the annotation of biochemical models) guidelines. BioModels now accepts large-scale submission of auto-generated computational models. With gradual growth in content over 15 years, BioModels currently hosts about 2000 models from the published literature. With about 800 curated models, BioModels has become the world’s largest repository of curated models and emerged as the third most used data resource after PubMed and Google Scholar among the scientists who use modelling in their research. Thus, BioModels benefits modellers by providing access to reliable and semantically enriched curated models in standard formats that are easy to share, reproduce and reuse.

## INTRODUCTION

Biological systems are complex in nature and their properties often emerge from a complex interaction between their components. Hence, predictive computational models are very useful to fully understand the behaviours of biological systems and generate hypotheses (1). Models of biological processes including, but not limited to cell signalling, metabolic and gene regulatory networks have been shown to divulge mechanistic insight into cellular regulation, disease formation and drug action. To address the growing need for a platform to exchange models, BioModels, a repository for computational models of biological and biomedical processes, was established in 2005 at the European Bioinformatics Institute (2). Since its inception, it has evolved significantly to become a core resource of the systems modelling community. In a recent survey of systems biologists (3) by the EU Infrastructure for Systems Biology in Europe consortium, BioModels was by far the most important repository for model deposition (33% of 157 respondents). In another recent survey among the scientists who use modelling in their research, BioModels emerged as the third most used data resource after PubMed and Google Scholar, and as the preferred model repository (4).

The rising popularity emanates from the FAIR (findable, accessible, interoperable and reusable) (5) principles of BioModels. All submitted models are assigned a unique and persistent identifier and annotated with metadata to make these depositions easily findable. Models along with their development history are accessible to the users both through a web interface and programmatically. Models submitted to BioModels are manually curated in due course to reproduce the simulation figures in the reference publications. Supporting interoperability, BioModels recommends deposition of models in standard formats such as SBML (6), CellML (7), COMBINE archive (8) and PharmML (9). Moreover, models encoded in proprietary formats are converted to community standards such as SBML during the curation process, thus facilitating the exchange of these models between different tools. Models are also semantically enriched with cross-references to standard data resources, ontologies, reference publication, etc. using machine-friendly controlled vocabularies. The clear provenance information and rich annotation along with our permissive CC0 license support model reuse. More importantly, users can be confident that the curated models have been independently verified to faithfully reproduce the results from their manuscript. In the past 15 years, both the content of BioModels and the technical infrastructure have continuously expanded to cater to the growing needs of the systems modelling community. In this article, we summarise recent developments as well as our future plans for BioModels.

## BIOMODELS CONTENT AND COVERAGE

As a part of an international initiative, BioModels was first established as a repository of curated quantitative kinetic models from the published literature (2). BioModels was an early resource to endorse standard formats for encoding models and provided kinetic models encoded in SBML (6). As a next step towards expansion, BioModels accepted submission of non-kinetic models, including constraint-based models of metabolic pathways, Petri net models, logic models, etc. Historically, only SBML and CellML (7) models were accepted for submission in the old BioModels platform. A brand new web infrastructure for BioModels based on JUMMP (https://bitbucket.org/biomodels/jummp-biomodels/) was developed and released in late 2017. The technical capabilities significantly improved after switching to the new infrastructure allowing BioModels to accept submissions and host models from diverse modelling approaches and formats (10).

**Figure 1.**
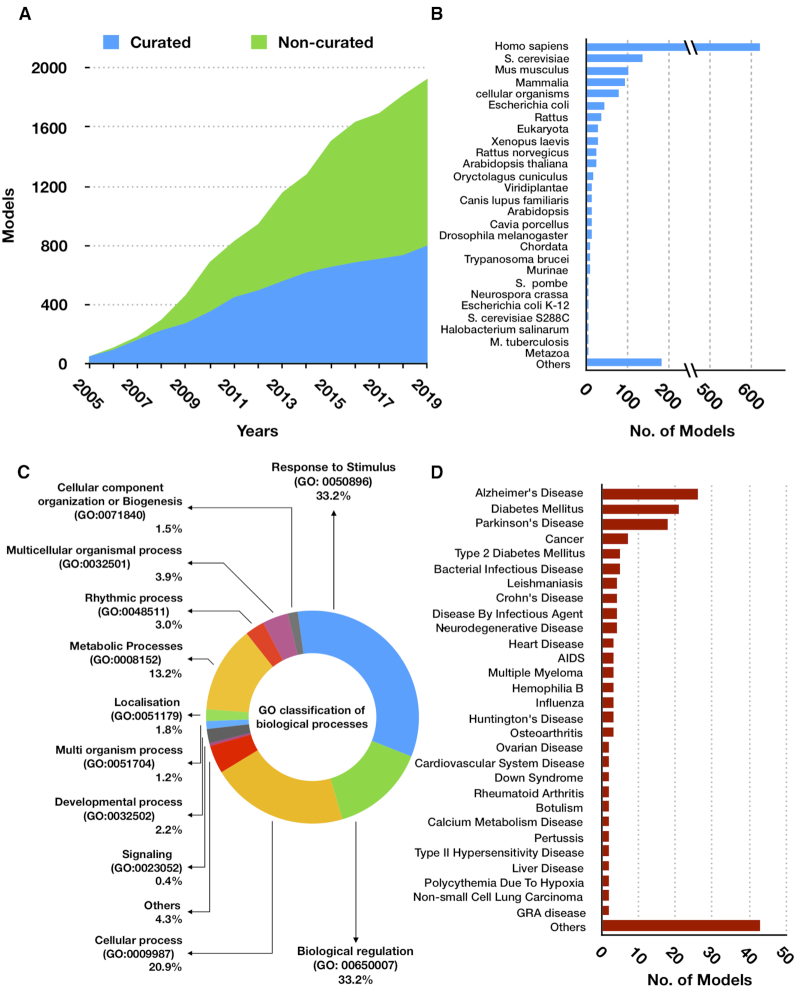
BioModels content and coverage. (**A**) The cumulative number of models in BioModels over the past 15 years in the curated and non-curated categories. (**B**) Distribution of models across taxons. (**C**) GO-based classification of models. (**D**) Distribution of models across diseases.

The number of models hosted in BioModels has gradually grown over the past 15 years. Currently, BioModels hosts about 2000 models from the published literature, among which about 800 models (Figure 1A) are manually curated and semantically enriched following MIRIAM (minimal information required in the annotation of biochemical models) guidelines (11). Models from over 60 different taxons are available; a majority (30%) of them are from *Homo sapiens* (Figure 1B). BioModels is also rich in coverage of diverse biological processes. A Gene Ontology (GO)-based categorisation of models reveals that about 50% of the models correspond to cellular processes (GO:0009987) and response to stimulus (GO:0050896) (Figure 1C). BioModels hosts a broad spectrum of disease models, including a considerable number emerging from the targeted curation of models of diabetes (12) and neurodegeneration (13) (Figure 1D). In addition to literature-based models, BioModels also hosts auto-generated models. This primarily includes two large-scale submissions in BioModels repository, namely, Path2Models (14) and patient-specific genome-scale metabolic models (15).

## MODEL CURATION AND ANNOTATION

**Figure 2.**
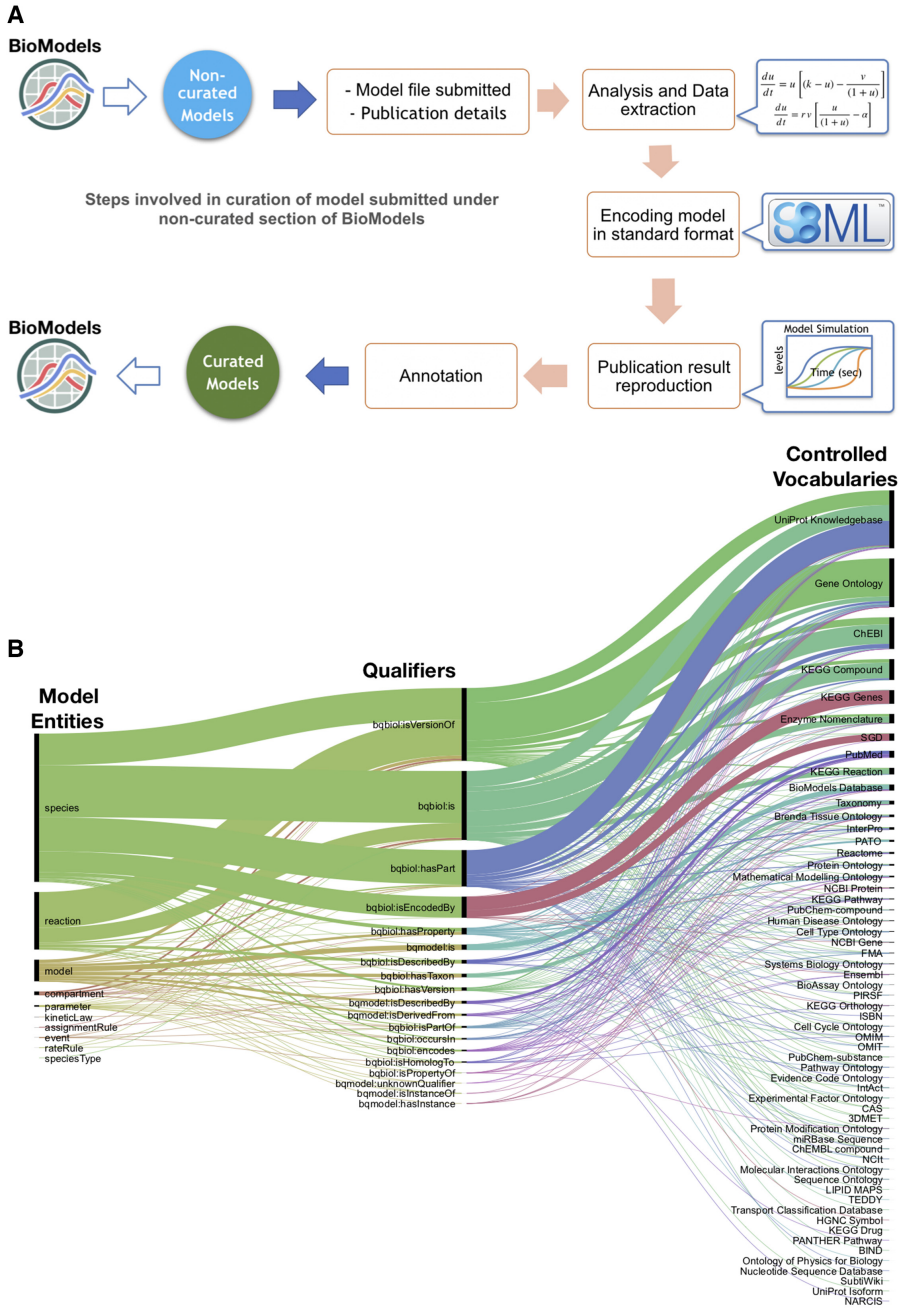
Model curation and annotation in BioModels. (**A**) Model curation workflow. (**B**) Semantic enrichment of models involves cross-referencing model entities (left) with controlled vocabularies and data resources (right) using COMBINE qualifiers (middle) that define the inter-relationship.

Many published models are lost for the community due to lack of sufficient characterisation of them to allow reuse (11). To address this, BioModels’ ethos since its inception has been to provide reliable, reusable curated models to the scientific community. The models are carefully curated; this requires encoding models in community standard formats such as SBML if submitted in other formats, cross-verification of model representation and ensuring that the numerical results of the simulation correspond to the reference publication (Figure 2A). The reproduced curation figure, together with comments from the curator on the simulation experiment and software used, is also provided to the users. For most curated models, BioModels offers the associated SED-ML file (16,17), a community standard for simulation experiment description, along with the COPASI (18) file used for simulation. To maximise the impact and reusability of the models, the MIRIAM guidelines were proposed (11) and the models are annotated following them in BioModels. Models are annotated with cross-references to controlled vocabularies such as GO, ChEBI, Mathematical Modelling Ontology, Systems Biology Ontology, Brenda Tissue Ontology and Experimental Factor Ontology, as well as data resources such as UniProt, Ensembl, NCBI Taxonomy, Reactome, etc. (Figure 2B).

BioModels’ search capability was built for leveraging the model annotation, enabling users to precisely search models and easily get an overview of the modelled biological phenomenon and the components. Model annotation can be valuable to combine models with either other ones or other datasets such as gene expression data (19). To enrich models from specific biological domains, targeted curation activity is performed. Our past efforts include curation of literature-based models of diabetes (13) and neurodegenerative diseases such as Alzheimer's and Parkinson's diseases (12), which added to our collection of disease models. The current focus is on the curation of blood coagulation, cell cycle and immuno-oncology models from the published literature and we have curated over 150 models pertaining to these areas. With 15 years of effort, BioModels has been growing as the world’s largest repository of curated mathematical models of biological processes.

## BIOMODELS FUNCTIONALITY

### Model submission

**Figure 3.**
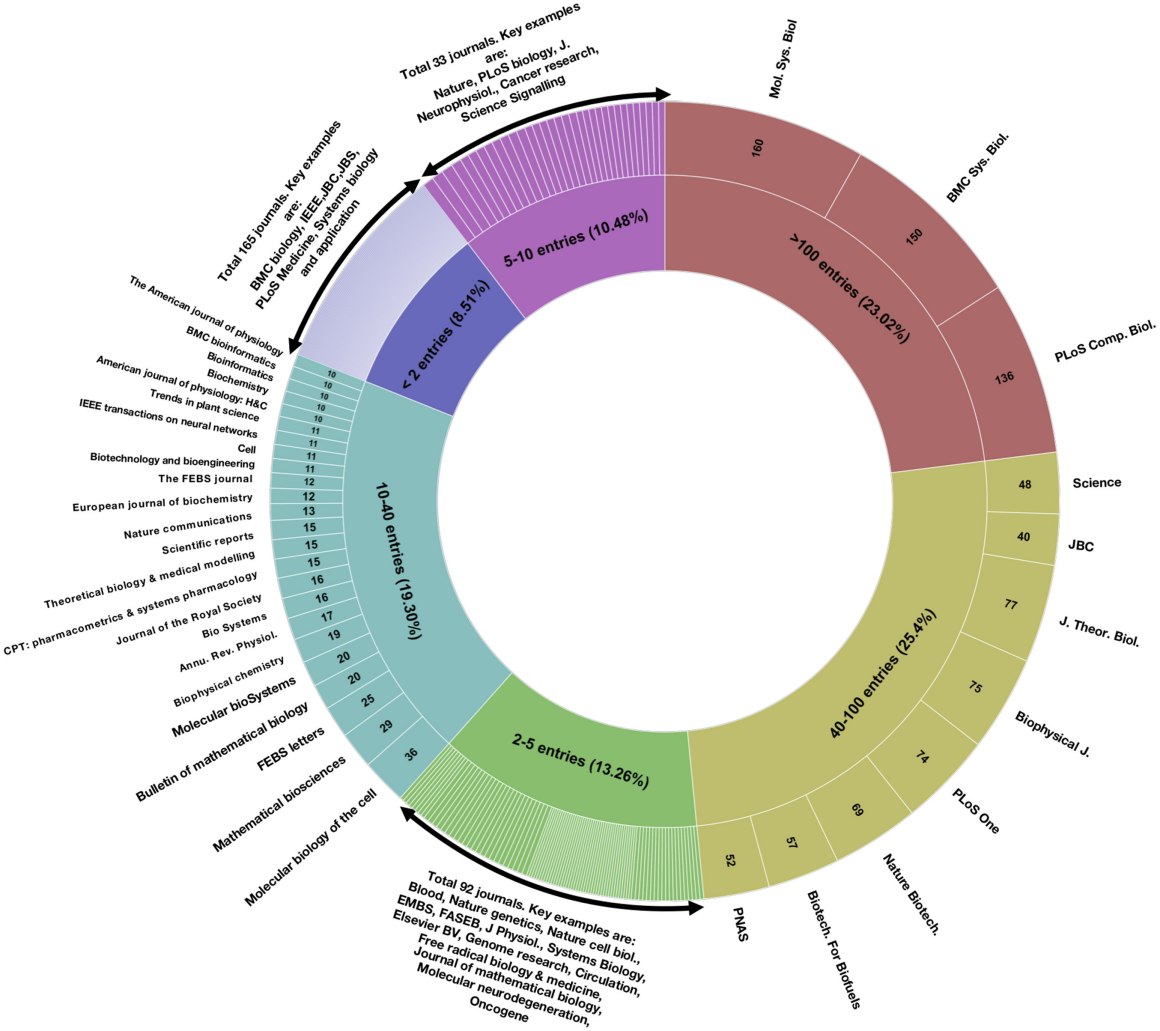
Provenance of models. BioModels hosts peer-reviewed models from over 300 journals.

BioModels was established to offer a platform to exchange published, peer-reviewed models between researchers across the globe. Models in BioModels stem from over 300 scientific journals specific to systems biology as well as general biology (Figure 3). Several journals recommend authors to submit models to BioModels (20). BioModels can also accept models published in bioRxiv.

**Figure 4.**
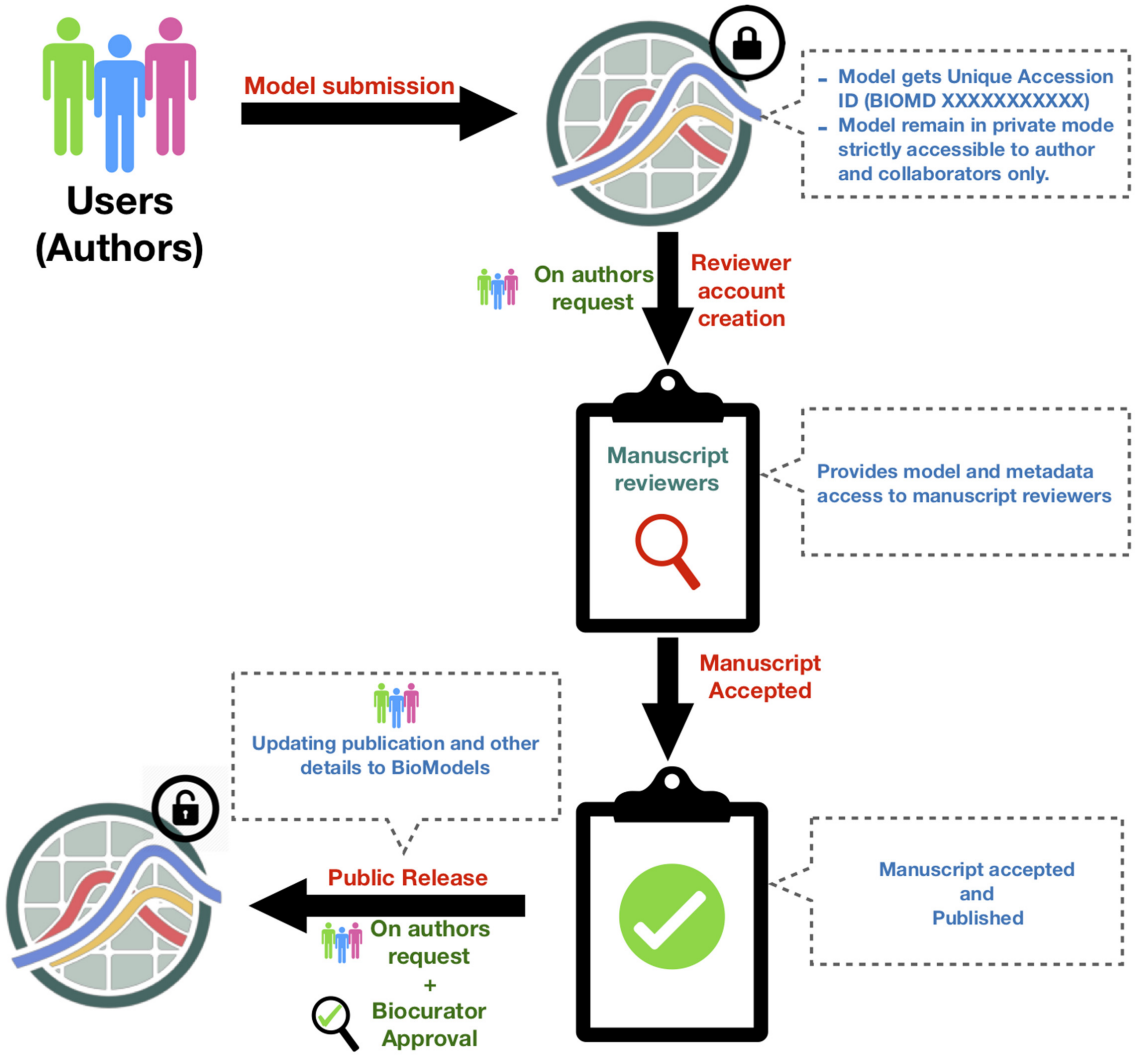
From submission to publication of a model in BioModels.

Modellers can submit their model to BioModels and include the unique model accession ID provided by BioModels in the manuscript before submission to the journal. The submitted model remains private and only accessible to the author and any collaborators who are given access to update and edit the model and the associated metadata. The version control system in BioModels transparently tracks changes to the model and associated files behind the scenes. BioModels also offers manuscript reviewers secure read-only access to a submission or set of related submissions in order to support the peer-review process. Upon either acceptance or publication of the manuscript, using the web interface, the author can update the publication details such as PubMed ID, abstract and authors’ list, and request the public release of the model (Figure 4).

### Model retrieval and search

The BioModels platform offers a sophisticated searching feature to facilitate easy search and retrieval of public models from the repository. Keyword-based queries can be combined with multiple facets to filter models based on curation status, model format, modelling approach, model organism, disease, GO-based annotation, and UniProt, ChEBI and Ensembl genes, to narrow down the search. The efficiency of the search is contingent on the annotations added to the model. Recently, we have introduced a tagging system in the BioModels platform to allow curators to add specialised labels (e.g. annotated, Path2Models, auto-generated) to the models, which can also be used to enhance search and filtering capabilities.

In addition to the faceted search, users can also browse models using the GO category chart. Furthermore, to facilitate easy search and retrieval of kinetic parameters, we have recently developed a new service, BioModels Parameters, that fetches and stores the data from the SBML models and displays them in a tabular format for quick access.

### Model display

The model display page provides a tab-based view of contents. The Overview tab displays information on the associated scientific article, including title, authors, abstract, etc., and the model level annotations, including the taxon, pathway, modelling approach, tissue type, tags, etc. The Files tab allows users to preview or download all model and associated files. The History tab provides a summary of public versions of the model and enables users to download a COMBINE archive of any version. The Curation tab displays the simulation figure reproduced by the curators and the associated public comments. Auto-converted alternative representations of the model, such as SBGN, Octave, PDF, BioPAX, etc., can be viewed and retrieved from the Export tab. In addition, the model display page also provides format-specific tabs. The curated SBML models will display a Components tab that visualises all the elements of the model, including species, reactions, parameters, kinetic rates, assignment rules, etc., along with their resolved annotations, if any, in a user-friendly tabular format.

### Tailored support for large-scale models

Path2Models, a large collection of over 140 000 models auto-generated from pathway resources, including KEGG, BioCarta, MetaCyc and SABIO-RK, was deposited into BioModels (14), as well as another collection of 6750 patient-specific genome-scale metabolic models (15) representing tumour growth in 17 cancer types. The latter was produced by combining RNA-seq data from individual patient samples with a generic human metabolic model (21). In response to the community’s desire to deposit submissions consisting of hundreds or thousands of models, we have devised and implemented a policy for handling them. Large-scale submissions will be bundled into groups of related models as COMBINE archive files. These model bundles can be seamlessly searched and retrieved either through a web browser or programmatically, just like all literature-based models hosted in BioModels. It is possible to either include or exclude them from the search results, and a batch download facility for such large-scale submissions is available through our FTP service.

We have reorganised the Path2Models submissions into 812 clusters based on their genus, using the models’ taxonomy cross-references. The resulting clusters consist of metabolic, non-metabolic and genome-scale metabolic models. For each deposition, the whole-genome metabolism model is considered the main model file of the submission, while all the other metabolic and non-metabolic models for the organisms in that genus are bundled into a COMBINE archive and made available as an additional file for that genus’ entry. Users accessing the Path2Models models that are now part of an additional file are transparently redirected to the corresponding representative model. This logical rearrangement strives to balance the need to make the information from these models accessible while allowing users to exclude them from the search results when necessary. Authors wishing to deposit large-scale submissions should contact the BioModels team for specific instructions.

### Programmatic access

Publicly available depositions hosted in BioModels can be searched and downloaded through our REST API (https://www.ebi.ac.uk/biomodels/docs/). To support the widest breadth of third-party tools without imposing any constraints on the programming language they are built in, the API is available over HTTP and it can return data in either XML or JSON—the two most widely used formats for data exchange. An extensive suite of well-established software tools, including CellDesigner (22), VCell (23), iBioSim (24), Tellurium (25), libRoadRunner (26) and SemGen (27), integrates the BioModels API into their business functionality, allowing their users to load models of interest remotely.

## BIOMODELS USAGE AND IMPACT

For the past 15 years, BioModels has been at the core of the systems modelling community with continuous growth in content and infrastructure to support the global user community. In 2018, every month on average over 23 000 unique hosts accessed BioModels approximately 816 000 times, downloading 232 GB data from BioModels.

BioModels offers a platform for enhanced visibility of published models. The curation of the model provides an extra layer of confidence in the model. The curated SBML models are ‘ready to use’ and they can be directly imported into any SBML simulation software to run simulations and reproduce results. As a result, these curated models are preferentially retrieved by our users, being downloaded 2.4 times more than the non-curated counterparts in 2018. As the curated SBML models are syntactically correct, they are used by SBML supporting software akin to test suites (24,28,29) to calibrate their software. Models from BioModels are also frequently used to develop novel computational approaches (30–32). The model curation in BioModels promotes reproducibility and is instrumental in supporting model reuse and repurposing (33).

## FUTURE DIRECTION

BioModels will continue to evolve in order to support the growing need of the systems modellers developing new approaches and standards. Some of the key short-term and long-term goals are the following. Currently, format-specific ‘Components’ tab in the model display page is available for SBML models and BioModels will invest resources to expand this functionality to other formats on demand from the community. We also plan to add support for programmatic submission of models, as this feature has been requested by systems model building software developers to directly submit newly built models. In addition, we will offer more control over the scope of search queries by allowing users to filter the auto-generated models.

At the COMBINE (http://co.mbine.org/) 2019 meeting (http://co.mbine.org/events/COMBINE_2019), we have initiated community discussion to jointly develop ModeleXchange, a lightweight, metadata-based infrastructure for the collaborative discovery of systems biology models and model components across independent repositories, including SynBioHub (34), Physiome Model Repository (35), JWS Online (36), ModelDB (37), BiGG (38), Open Source Brain (39), Center for Reproducible Biomedical Modeling (https://reproduciblebiomodels.org/) and V-Cell (23). ModeleXchange will aim to provide the global user community with a single entry point for model discovery and deposition, backed by a distributed infrastructure.

Multi-scale modelling—building models spanning a broad spatial scale from molecules to tissues to organisms and timescales from microseconds to days to years—is gaining interest in the community. Hence, in the long run BioModels will continue to build technical competence to facilitate multi-scale model building, dissemination and storage. A mirror site for BioModels is available at Caltech (http://biomodels.caltech.edu) in order to improve geographical load distribution and thus minimize latency. We are now aiming to make the BioModels platform easily installable in a cloud-based environment, in order to provide a fast and reliable service to our users across the globe. Furthermore, to bring mathematical modelling to the reach of experimental biologists, BioModels will strive to build new tools to enable analysis of multi-omics data such as RNA-seq, proteomics and genetic variation data with curated quantitative models.

The curation of models has been pivotal in making BioModels a successful repository; BioModels will continue its curation effort and enhance functionality to fully support multiple modelling approaches. BioModels will remain committed to the ethos of FAIR data sharing in the field of systems modelling.
